# Potential Novel Strategies for the Treatment of Dental Pulp-Derived Pain: Pharmacological Approaches and Beyond

**DOI:** 10.3389/fphar.2019.01068

**Published:** 2019-09-18

**Authors:** Christina M. A. P. Schuh, Bruna Benso, Sebastian Aguayo

**Affiliations:** ^1^Centro de Medicina Regenerativa, Facultad de Medicina Clínica Alemana-Universidad del Desarrollo, Santiago, Chile; ^2^School of Dentistry, Faculty of Medicine, Pontificia Universidad Catolica de Chile, Santiago, Chile; ^3^Department of Physiology, Faculty of Medicine, Universidad Austral de Chile, Millennium Nucleus of Ion Channels Associated Diseases (MiNICAD), Valdivia, Chile

**Keywords:** dentistry, pain, dentin, dental caries, regenerative medicine, pharmacology, collagen, endodontics

## Abstract

The diagnosis and management of pain is an everyday occurrence in dentistry, and its effective control is essential to ensure the wellbeing of patients. Most tooth-associated pain originates from the dental pulp, a highly vascularized and innervated tissue, which is encased within mineralized dentin. It plays a crucial role in the sensing of stimuli from the local environment, such as infections (i.e. dental caries) and traumatic injury, leading to a local inflammatory response and subsequently to an increase in intra-pulp pressure, activating nerve endings. However, thermal, chemical, and mechanical stimuli also have the ability to generate dental pulp pain, which presents mechanisms highly specific to this tissue and which have to be considered in pain management. Traditionally, the management of dental pulp pain has mostly been pharmacological, using non-steroidal anti-inflammatory drugs (NSAIDs) and opioids, or restorative (i.e. removal of dental caries), or a combination of both. Both research areas continuously present novel and creative approaches. This includes the modulation of thermo-sensitive *transient receptor potential cation channels* (TRP) by newly designed drugs in pharmacological research, as well as the use of novel biomaterials, stem cells, exosomes and physical stimulation to obtain pulp regeneration in regenerative medicine. Therefore, the aim of this review is to present an up-to-date account of causes underlying dental pain, novel treatments involving the control of pain and inflammation and the induction of pulp regeneration, as well as insights in pain in dentistry from the physiological, pharmacological, regenerative and clinical perspectives.

## Introduction

Despite many advances in the fields of diagnosis, material sciences and therapeutics, oral diseases continue to burden millions of people worldwide, causing a significant impact on both health costs and patient quality of life (Hall-Stoodley et al., 2004; Logan and Brett, 2013; Römling et al., 2014). According to the Global Burden of Disease Study 2016, it is estimated that more than 3.5 billion people worldwide suffer from oral diseases, with 2.4 billion of those cases being dental caries (Vos et al., 2017). Furthermore, it is also estimated that 743 million people are affected by periodontal disease, a chronic progressive weakening of the supporting structures of the tooth that leads to tooth loss and dysfunction (Tonetti et al., 2017). Despite the fact that these oral diseases display a wide variety of symptoms, many patients seek dental advice due to the presence of pain in the mouth and/or facial region. Therefore, the diagnosis and management of orofacial pain is an essential need for dentists to ensure the wellbeing of patients, as well as to determine the most appropriate treatment plan for each clinical situation.

In general terms, pain can be defined as an “unpleasant sensory and emotional experience that is associated with actual or potential tissue damage or described in such terms” (Gebhart, 2000). Thus, it is a subjective appreciation that varies between individuals, which can make its clinical diagnosis challenging. In dentistry, pain is a multifactorial experience that also includes strong emotional and previous-experience components (Sessle, 1986). There are many origins of pain in the orofacial region; however, the most frequent pain is the one initiating from within the tooth and dental pulp (Estrela et al., 2011), which can be triggered by a wide and diverse range of stimuli including hot and cold temperatures, air puffs, sugar consumption, and mechanical pressure. Orofacial pain originating from structures surrounding the tooth (such as periodontal tissue, oral mucosa and alveolar bone) will not be covered in this review.

### Tooth Anatomy and Its Relevance in Dental Pain Perception

**Figure 1:**
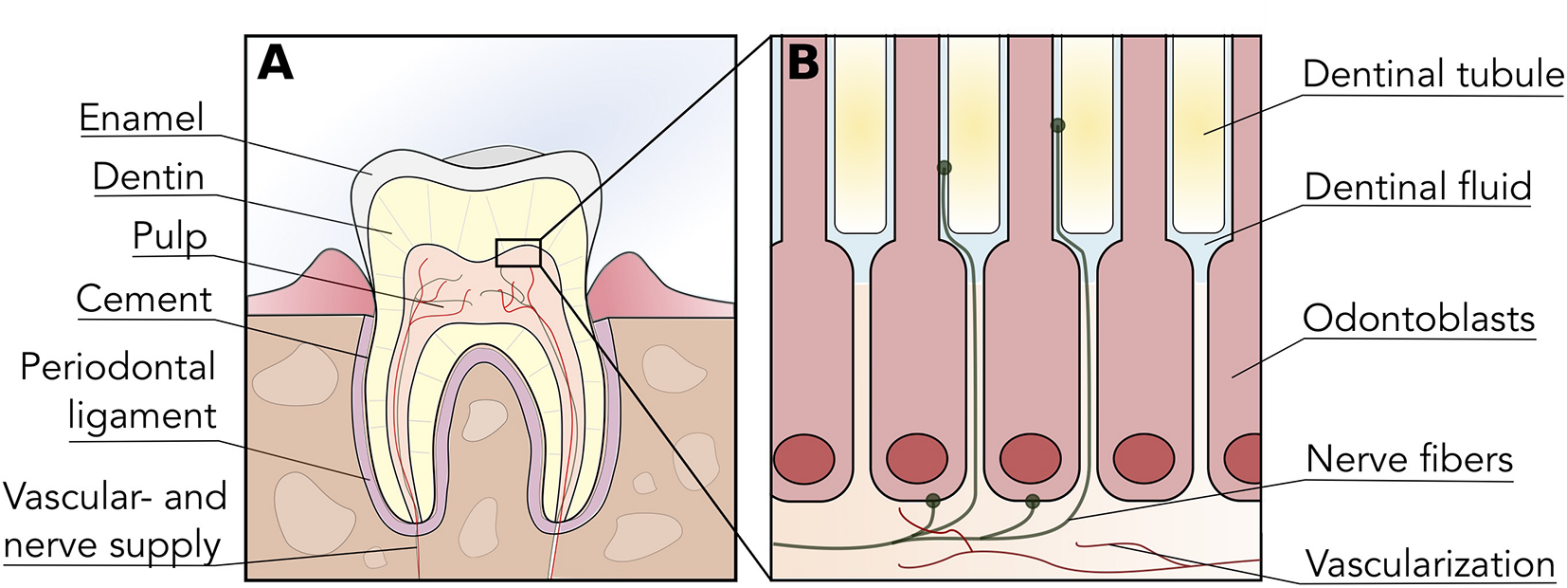
Tooth and dental pulp anatomy. **(A)** Diagrammatic representation of a tooth cross-section illustrating the organization of enamel, dentin, cement and dental pulp within the tooth structure. The dental pulp is vascularized and innervated through the root apex, which provides nociception *via* afferent trigeminal nerves. **(B)** The odontoblast layer is located in the interphase between dentin and dental pulp, with prolongations extending into dentin *via* dentinal tubules. Sensory nerve fibers penetrate the odontoblast layer and enter the initial portion of dentinal tubules, thus permeability or alterations in dentin can trigger nociception.

Teeth are highly complex organs located within the oral cavity. They are constituted by three mineralized tissues known as enamel, dentin, and cement, which surround the un-mineralized tissue known as dental pulp (**Figure 1A**). Mineralized tissues of the tooth are mostly comprised of hydroxyapatite crystals with varying amounts of organic content (e.g. type-I collagen, other proteins), and their main functions are to provide structural integrity to the tooth and to protect the pulp from environmental injury (Shahmoradi et al., 2014). Enamel is the most mineralized tissue in the body; therefore, it plays a central role in mastication and in protecting dentin and pulp from environmental injury. Cement, on the other hand, is present on the surface of the root, and is involved in the anchoring of the tooth to the alveolar bone by means of the periodontal ligament.

Dentin is a mineralized dental tissue contained within enamel and cementum, constituted by hydroxyapatite (70% weight), organic material (20% weight) and water (10% weight) (Goldberg, 2011). The organic material is mostly comprised of a matrix of type-I collagen, and its presence is crucial for the structural stability as well as the elasticity of the tooth (Butler and Ritchie, 1995; Bertinetti et al., 2015). Dentin is maintained by the cellular prolongations of odontoblasts, which are nested within dentinal tubules- channels extending from the dental pulp until the enamel-dentin junction (Arana-Chavez and Massa, 2004) (**Figure 1B**). These tubules are flooded with dentinal (interstitial) fluid; thus, dentin receives nutrients by direct perfusion from the dental pulp. Most importantly, as dentin is strongly interconnected with the dental pulp and its innervation, it is crucial in the process of dental pain perception.

Interestingly, the initial portion of the dentinal tubules contains pulp nerve endings and thus dentin exposed to the oral cavity (i.e. by caries or trauma) can actively respond to environmental stimuli and trigger nociception (**Figure 1B**). Stimulatory sources that can directly or indirectly affect the dental pulp include chemical irritation, dental caries, infiltration of bonding materials, trauma and orthodontic movements, among others (Tikhonova et al., 2018). This phenomenon is also known as *dentinal hypersensitivity*, as seemingly innocuous stimuli can generate an exaggerated pain response in patients (Miglani et al., 2010). Nerve endings located in the initial portion of dentinal tubules and dental pulp are mainly constituted of A-delta and C fibers—afferent endings of the trigeminal nerve—that can be triggered by both mechanical and thermal stimuli (Jain et al., 2013).

There are three main hypotheses regarding the mechanisms behind dentinal hypersensitivity. The first hypothesis, also known as the *neural theory*, states that nerve endings within dentinal tubules are directly able to respond to external stimuli. Nociceptive temperature-sensitive receptors such as TRPV1 and TRPA1 are believed to be involved in pain transduction from hot and cold thermal stimuli (Chung et al., 2013). The second hypothesis or *hydrodynamic theory* proposes that thermal and mechanical stimuli, such as air spraying, can exert pressure changes that cause fluid movement within the dentinal tubules (Andrew and Matthews, 2000; Shibukawa et al., 2015). This movement is believed to trigger A-delta and C fibers, although the presence of specialized mechanoreceptors in these fibers is still debatable. Finally, the *odontoblast transducer theory* states that odontoblast cells themselves are responsible for pain perception. Expression of TRPV channels in odontoblasts as well as their direct excitability support this theory (Won et al., 2018).

Additional to dentinal hypersensitivity, dental pain can also be a consequence of localized inflammation within the dental pulp. This response is at times exacerbated due to the lack of compliance given by the surrounding mineralized tissues, and therefore minor inflammatory changes can result in exaggerated pain perception. Known pro-inflammatory mediators involved in pulpal pain include substance P, calcitonin gene-related peptide, histamine, and cytokines (Caviedes-Bucheli et al., 2008, Caviedes-Bucheli et al., 2011; Sessle, 2011). These molecules are responsible for sensitizing nerve endings as well as increasing intra-pulp pressure by virtue of increasing vascular supply to the dental pulp (Rechenberg et al., 2016). Furthermore, inflammatory mediators released as part of the response system act in the production of vascularized alterations mediated by different receptors such as those from toll-like receptor (TLR) variants 2 and 4 (Nakanishi et al., 2011). It is believed that TLR-2 positive cells are present in the early phases of regulation (including the first 3 h after stimulus), which recognize different pathogen-derived molecules such as triacyl lipopeptides from bacteria and mycobacteria, peptidoglycan, and lipoteichoic acid of gram-positive bacteria and zymosan from fungi (Park et al., 2015; Rechenberg et al., 2016). On the other hand, TLR4 has a central role in the response to co-stimulatory molecules originating from gram-negative bacteria, such as lipopolysaccharides (LPS) (Nakanishi et al., 2011; Park et al., 2015). Considering TLR and the release of pro-inflammatory cytokines like IL-8, IL-6 and chemokines, the succession of stimulatory events can lead to a painful and hyperalgesic process within the dental pulp (ElSalhy et al., 2013). The wide range of factors and stimuli involved leads many to describe the dental pulp as an innervated tissue associated with pain transduction mechanisms that have yet to be fully explained (Mutoh et al., 2007).

### Dental Caries and Its Relevance in Dental Pain

There are several known causes responsible for the onset of dental pain including infection, trauma, dental treatment and chemical injury (Alonso-Ezpeleta et al., 2012; Jain et al., 2013; Khan et al., 2019). These situations share the common factor of either exposing dentin or dental pulp to the environment and/or generating an inflammatory process within the dental pulp, initiating dentinal hypersensitivity, and/or inflammatory pain. Nevertheless, in the clinical setting, the most common source of dental pain is due to dental caries.

Dental caries (also known as tooth decay) affects the vast majority of the population and is one of the main causes of tooth loss in patients of all ages (Petersen et al., 2005; Kassebaum et al., 2014; Dye et al., 2015). Worldwide, recent data shows that most adults have been affected by dental caries, and therefore this pathology represents a large economic burden to individuals and healthcare providers. The Global Burden of Disease Study 2013, reporting data on 301 diseases and injuries, concluded that tooth pain resulting from caries affected over 200 million worldwide, and was the fifth commonest acute condition observed overall (Global Burden of Disease Study, 2013; Rice et al., 2016).

Caries is a biofilm-mediated multifactorial infectious disease characterized by the loss of mineral from the tooth surface due to bacterial colonization, which can affect all mineralized tissues in teeth (Pitts et al., 2017). Although caries pathogenesis is highly complex and multifactorial, it is believed to be initiated by species such as *Streptococcus mutans* and *lactobacilli*, which induce surface demineralization by the production of extracellular acids that destroy the mineralized matrix of the tooth (Beighton et al., 2010; Krzyściak et al., 2014; Wassel and Khattab, 2017). The process of caries formation starts on the surface of the outer layers of the tooth, and if not treated it progresses into the underlying dentin layer (Pitts et al., 2017). Caries can also affect the root portion of teeth once this area becomes exposed to the oral environment. This is common in elderly people suffering from gingival recession, and thus these patients are at higher risk of developing root caries lesions (Wierichs and Meyer-Lueckel, 2015). Therefore, dental caries can trigger dental pain in a two-fold manner: by exposing dentin to the environment with subsequent onset of dentinal hypersensitivity, and by generating a localized pulp inflammation in response to bacterial invasion and bacterial molecules (such as bacterial peptides and LPS).

Pain as a consequence of dental caries is a known occurrence in the clinics, which is also supported by many investigations demonstrating the association between caries and pain in children and adults (Reid et al., 2003; Nomura et al., 2005; Bastos et al., 2006). In children, pain generated by caries has also been associated to reduced child weight, growth, and quality of life (Sheiham, 2006). Several studies have provided insight into the mechanisms behind how caries can trigger dental pain within the dental pulp. Firstly, the progressive demineralization generated by caries dissolves the outer layers of the tooth and exposes dental pulp nerve endings to environmental stimuli, triggering dentinal hypersensitivity. However, there is also evidence suggesting bacterial toxins are able to permeate into the dental pulp *via* dentinal tubules and initiate an inflammatory response within odontoblasts and the surrounding tissue (Farges et al., 2015). Durand et al. (2006) showed that lipoteichoic acid (LTA), an important bacterial wall molecule, increased the expression of chemokines in odontoblasts *via* upregulation of TLR 2. Similarly, Farges et al. (2011) observed increase in pro-inflammatory IL-6 production in odontoblasts upon LTA stimulation. Furthermore, cyclooxygenase (COX) has been shown to be associated to tissue inflammation on several levels. In inflamed tissues there is an increase in the biosynthesis of prostaglandins, which are generated by COX isoenzimes metabolizing arachidonic acid (Ricciotti and FitzGerald, 2011). Also, while COX-1 is constitutively expressed in many tissues, COX-2 is promoted by inflammatory stimuli and is directly involved with inflammatory pain generation (Dubois et al., 1998). Within this context, Petrini et al. (2012) demonstrated an increase of prostaglandin E2 in teeth with reversible dental pulp inflammation compared to normal controls. In addition, the penetration of bacterial cells into the dental pulp may also be an important stimulus for nociception, as Chiu et al. (2013) demonstrated that bacteria can directly activate nociceptors in a mice model. Therefore, it is necessary to remove all caries-infected dental tissue as a previous step before beginning tooth restoration, in order to completely resolve inflammation in the dental pulp. However, in cases of extreme pulpal inflammation or when the caries lesion enters into the pulp chamber, dental pulp necrosis may occur. In these cases, the dental pulp must be completely removed by means of a root canal procedure (pulpectomy) and replaced by thermoplastic material.

Furthermore, Rodd and Boissonade (2000) demonstrated an increase of substance P in carious teeth compared to healthy samples, and its expression was further increased in painful samples compared to asymptomatic teeth. The levels of another neuropeptide, neuropeptide Y, was also found to be elevated in teeth affected by caries compared to controls (El Karim et al., 2006) TRPV-1 expression was also increased in carious teeth compared to controls, and vascular expression of TRPV-1 within the dental pulp was associated to increased dental pain (Morgan et al., 2005). Recently, Hall et al. (2016) demonstrated an upregulation of TNF-α in patients with caries, which was even more pronounced in patients with dental pain.

## Current Pharmacological Approaches for Dental Pain Treatment

The therapeutic management of sensitive and painful regions is one of the most relevant aspects in the dental practice due to its direct influence on patient quality of life (Murray et al., 1996). Current management of pain deriving from inflammation utilizes several approaches including local intervention and adjuvant pharmacological treatments (Ward, 1974; Dal Secco et al., 2006). Local interventions, particularly dental restorations, aim to isolate the dental pulp and dentin from environmental stimuli triggering hypersensitivity as well as remove caries lesions to resolve underlying pulpal inflammatory process. On the other hand, pharmacological treatment contributes to the promotion of analgesia, with frequently used drugs being nonsteroidal anti-inflammatories (NSAIDs), opioids and NMDA receptor blockers (Chou and Huffman, 2007). Considering that the origin of pulpal pain can occur at different points during treatment (i.e. preoperative, perioperative, and postoperative), the drug strategy must be chosen according to the individual conditions of each clinical case (Primosch et al., 1993; Vanegas et al., 2010). For example, analgesic drug treatment can begin before intervention (known as pre-emptive analgesia) with the aim of inhibiting the inflammatory process prior to dental restoration in asymptomatic patients (Ong et al., 2005). As several investigations have discussed conventional pharmacological management of dental pain in the past (Hargreaves and Abbott, 2005; Becker, 2010; Moore and Hersh, 2014; Berlin et al., 2018), this review will only provide a brief summary of current treatment options utilized in clinics.

Currently, the first line of drugs for the treatment of dental-derived pain are acetaminophen and NSAIDs (Moore et al., 2018). Both have been shown to be essential in the management of minor to moderate post-operative pain in dentistry (Bailey et al., 2013) and proven to be safe and effective, having them as the most favored options among treatments available in the market (Ong et al., 2005). NSAIDs have been demonstrated to inhibit the action of COX-1 and COX 2, thus are important in controlling inflammatory pain in a wide range of tissues (Brune and Patrignani, 2015). On the other hand, the exact mechanism of action for acetaminophen in humans remains unclear, although some authors believe it has an effect on a splice variant of COX-1 (previously believed to be the COX-3 isoenzyme) (Graham and Scott, 2005; Bertolini et al., 2006; Sharma and Mehta, 2013). In conjunction with opioids, acetaminophen and NSAIDs such as ibuprofen have been demonstrated to alleviate moderate to severe postoperative pain (Ashley et al., 2016) as well as acute dental pain (Chang et al., 2001; Bailey et al., 2013). Pain treatment of vulnerable patient groups such as elderly or patients with gastrointestinal issues is challenging, however there is supportive evidence for the use of acetaminophen alone or in combination with codeine (Ong et al., 2007; Ganzberg, 2018). Studies show that associating NSAIDs with opioids may have the additional benefit of controlling post-operative pain (**Table 1**), however, selective COX-2 inhibitors have been proven to be more effective (Chang et al., 2001; Korn et al., 2004). Additionally, special considerations should be taken to reduce opioid overprescribing and abuse, especially with young patients and patients that can be managed solely with NSAIDs (Noble et al., 2010).

**Table 1:** Pharmacological treatment possibilities to control post-operative pain in dentistry with opioids alone and in different combinations.

		Authors*	Study design	Sample size	Pharmacological treatment groups	Outcome instrument	Postoperative measurement	Postoperative assessment of pain	Study conclusions
**Opioid alone**	**Tramadol**	(Mehrvarzfar et al., 2012)	Randomized clinical trial, double-blind and placebo-controlled	100	Placebo Tramadol 100 mg Acetaminophen 325 mg +Ibuprofen 200 mg+ Caffeine 40 mg (Novafen) Naproxen 500 mg	10-point VAS	Placebo 3.2 (2.6–3.9) Tramadol 2.2 (1.2–3.1) Novafen 0.4 (0.1–0.8) Naproxen 0.7 (0.3–1.1)	24 h	A single oral dose of Naproxen, Novafen and Tramadol taken immediately after dental treatment reduced postoperative pain following pulpectomy.
	**Codeine**	(Sunshine et al., 1983)	Randomized clinical trial, double-blind and placebo-controlled	120	Placebo Propiram Fumarate 50 mg Codeine Sulfate 60 mg	4-point rate scale none = 0; mild = 1; moderate = 2, severe = 3	Placebo 1.27 (0.25) Propiram Fumarate 2.11 (0.22) Codeine 1.72 (0.23)	6 h	Propiram fumarate 50 mg is an effective oral analgesic similar to codeine sulfate 60 mg, with the possibility of a longer effect.
**Opioid combination**	**Tramadol/Acetaminophen**	(Edwards et al., 2002)	Systematic- review with meta-analysis	1376	Acetaminophen 650 mg Ibuprofen 400 mg Tramadol 75 mg Tramadol 75mg/acetaminophen 650 mg	NNT (95% CI) PGE**	4.5 (3.6–6.1) 2.7 (2.3–3.3) 11 (6.9–26) 3.0 (2.5–3.7)	8 h	Overall, this meta-analysis demonstrated analgesic superiority of the combination drug over its components, without additional toxicity.
	**Codeine/Acetaminophen**	(Chang et al., 2001)	Randomized clinical trial, double-blind and placebo-active controlled	393	Placebo (1:6:6, respectively) Rofecoxib 50 mg Codeine 60 mg/Acetaminophen 600 mg	TOPAR6***	Placebo 3.4 (1.0–5.8) Rofecoxib 12.4 (11.3–13.4) Codeine/Acetaminophen 7.0 (5.9/8.0)	6 h	In this study of moderate to severe postoperative dental pain, the analgesic efficacy of Rofecoxib 50 mg was greater than that of codeine/acetaminophen, with a lower incidence of adverse events and nausea.
	**Oxycodone/Acetaminophen**	(Korn et al., 2004)	Randomized clinical trial, double-blind and placebo-active controlled	212	Placebo (1:3:3, respectively) Rofecoxib 50 mg Oxycodone 5mg/Acetaminophen 325 mg	GLOBAL24^#^	Placebo 0.3 (0.2) Rofecoxib 2.1 (0.2) Oxycodone/Acetaminophen 1.3 (0.2)	24 h	The superior efficacy of Rofecoxib 50 mg compared to oxycodone/acetaminophen 5/325 mg support the use of Rofecoxib for the treatment of acute post-surgical pain.

*All studies included in this summarized tables were checked for GRADE criteria; **PGE- Patient Global Evidence; ***TOPAR6- pain relief over 6 h; ^#^GLOBAL24 - Global assessment of treatment at 24 h.

Multimodal drug therapy is achieved by combining drugs with complementary mechanisms of action with the aim of reducing postoperative pain (Brooks et al., 2017; Matute Crespo and Montero Matamala, 2017). When treating moderate to severe pain, combination pharmacotherapy may be an effective alternative to treat pain and improve patient recovery times (Ganda, 2008). Originally, multimodal analgesia was advocated for use in ambulatory anesthesia combining NSAIDs and opioid analgesics, with or without anesthetic infiltration (Devin and McGirt, 2015). Combining oral or IV acetaminophen with selective or non-selective cyclooxygenase-2 inhibitors (COXIBs) has also demonstrated to be a safe option for pain treatment (Ong et al., 2007). These drugs can be administered *via* different routes, however special considerations must be taken into account when treating high-risk patient groups such as the elderly (Becker, 2010), as there are still conflicting reports in the literature regarding their safety and tolerability in these patients (Ganda, 2008). Overall, dental practitioners should make clinical decisions based on optimal described dosages, with non-opioid drugs as the primary treatment option (Becker, 2010). Nevertheless, in the case of persistent acute pain, short-acting opioid drugs may be an interesting alternative for treating this group of patients (Becker and Phero, 2005).

Despite the widespread use of acetaminophen, NSAIDs and opioids, and the benefits they provide for dental pain management, it is important to address several limitations of these drugs. Drug selection criteria is directly related to patient treatment needs and potential risk factors (such as cardiovascular disease), as there is a high number of potential interactions between commonly used NSAIDs and other medications (Ganzberg, 2018). As a general recommendation, patients must be evaluated for safety and possible adverse effects before taking NSAID treatment into consideration, especially in high-risk groups such as medically compromised patients (Wongrakpanich et al., 2018). A number of side effects for NSAIDs and opioids have been reported, such as gastrointestinal problems, nausea, and constipation (Benyamin et al., 2008; Nagi et al., 2015; Ganzberg, 2018). Furthermore, dependence to some of these drugs is currently a critical issue for public health in the US and worldwide, particularly opioid addiction due to misuse and excessive prescription (Nack et al., 2017; Jones et al., 2018). Therefore, several innovative approaches are currently being pursued as alternatives to conventional pharmacological pain management therapy in dentistry, including novel drug targets and regenerative and non-invasive medical approaches.

## Future Trends and Novel Insights Into the Pharmacological Treatment of Pain in Dentistry: Unveiling TRPV1 as a Drug Target

As previously stated, dental pulp pain has its origin in the stimulation of the nerve fibers acting as a complex defense mechanism against external factors (Magloire et al., 2010; Solé-Magdalena et al., 2018). When dental pulp suffers an injury, the nociceptive neurons initiate and increase a process known as neurogenic inflammation (Chung et al., 2013; Mizumura and Murase, 2015). Within this context, transient receptor potential (TRP) proteins are known to play an important role in dental nociception. TRP proteins are a large family of cation-permeable ion channels (Latorre et al., 2007; Nilius and Owsianik, 2010) sensitive to electrical, chemical, mechanical, and thermal stimuli, and many of them act as cell receptors involved in environmental detection (Morales-Lázaro et al., 2013). Current research links TRP proteins to hereditary neuropathies, neuronal disorders, and other TRP channel-associated channelopathies (Rosenbaum and Simon, 2007). The group of temperature-activated TRP channels (thermoTRPs) is composed of different sub-families (i.e. vanilloid, melastatin, ankiryn, and canonical) which are important for the detection and integration of peripheral sensory input (Caterina, 2007). Therefore, the understanding of how certain ligands modulate close-open configurations of thermoTRP channels could significantly facilitate the pharmaceutical design and elaboration of TRP drug modulators (Rosenbaum and Simon, 2007; Steinberg et al., 2014; Yang and Zheng, 2017).

The discovery of TRPV1 channels and their role in the signaling process and temperature-mediated nociception has enabled progress in strategies for managing and treating pain (Caterina et al., 1997; Rosenbaum and Simon, 2007; Julius, 2013). TRPV 1-4 thermal detectors are expressed in sensory nerves, transducing both proprioceptive and nociceptive information and providing information about environmental as well as body temperature to the central nervous system (Baez et al., 2014). Agonist-induced activation of TRPV1 channels has been critical to their identification and functional description (Matta and Ahern, 2007; Gao et al., 2016). At the molecular level, the canonical agonist capsaicin binds to the channel with high affinity (< 0.7 interaction score) and enables discrimination of TRPV1 from other vanilloid subtype channels (Yang and Zheng, 2017). Several vanilloid-related compounds are also able to induce transition to the open state with high affinity; interestingly, most of them are compounds of natural origin or minor derivatives of the natural chemical structure (Norman et al., 2007; Cui et al., 2016). Moreover, the gating mechanism in TRP channels as well as traffic to the plasma membrane is strongly regulated by phosphoinositides and their sub-products [diacylglycerol (DAG), inositol triphosphate (IP3)], polyunsaturated fatty acids (PUFAs) as well as metabolites generated by COX, LOX and CYP enzymes (Rosenbaum and Simon, 2007). To date, many compounds capable of inhibiting TRPV1 have been discovered such as resiniferatoxin (agonist), GRC6211 (antagonist), and QX-314 (TRPV1 permeable Na+ channel blocker); however, none of them is available yet for clinical therapeutics (Meotti et al., 2014). As allosteric control by lipid binding adds pharmacological properties to TRPV1, here the intention is to discuss the modulation of TRPV1 cellular activity by antagonists in the context of the pharmacological basis of oral pain sensation caused by inflammatory conditions (Steinberg et al., 2014).

The study and development of composites capable of acting on TRPV1 receptors have evolved greatly over time and capsaicin has been identified as the selective agonist ligand (Cheung et al., 2008; Gregorio-Teruel et al., 2014). The mechanism through which capsaicin exerts its function is known as tachyphylaxis, a rapid desensitization phenomenon associated to pore opening of cation channels permeable to sodium and potassium (Gavva et al., 2004; Ohbuchi et al., 2016). It is dependent on the exposure of the ligand to the channel, i.e., that the influx of Ca2+ ions has a protective feedback mechanism that presents a reduced response to repeated exposure (Brauchi and Orio, 2011; Yang and Zheng, 2017). The reversible effect of channel desensitization was also observed in situations of overdose and epidermal degeneration of nerve fibers (Rosenbaum and Simon, 2007; Cheung et al., 2008).

In clinics, capsaicin-derived formulations to treat severe and chronic pain are available in a drug delivery system known as transdermal patches (Carnevale and Rohacs, 2016). Formulations with a stable release of 8% capsaicin (80 mg per gram of adhesive) as a dermal patch have been approved for use on neuropathic pain in the US and Europe, and can be used at multiple sites according to patient needs (Trevisani and Szallasi, 2010). Randomized clinical trials in diseases like diabetes, traumatic tissue injuries, herpes zoster infections, and cancer among others, have proven the clinical safety and efficacy of the formulation in terms of improving patient quality of life (Kulkantrakorn et al., 2013).

Capsaicin has also been tested in association with other drugs such as local anesthetics (Gerner et al., 2008). Among these is the quaternary lidocaine derivate QX-314, which displays the therapeutic advantage of prolonging the anesthetic effect 10-times when compared to lidocaine hydrochloride (Nakagawa and Hiura, 2013). Ries et al. (2009) utilized capsaicin as a V1 agonist, enabling QX-314 to reach its intracellular binding site in the sodium channel. This scenario suggests that QX-314 in the presence of capsaicin can produce selective blocking mediated by TRPV1 (Binshtok et al., 2007). This pharmacological association was tested *in vivo* for an inferior alveolar nerve block, where a selective, long-lasting effect on sensory perception was noted without affecting motor areas (Lim et al., 2007). The above-described selective and long-lasting activity could be ideal in future formulations for the treatment of odontalgias caused by inflammatory processes, as well as in painful episodes caused by temporomandibular joint disorders (Kim et al., 2010a). Also, it demonstrates the potential of capsaicin and vanilloid-1 derivatives in the clinical management of pain associated with frequent episodes of pulpal inflammation in dentistry (Hossain et al., 2019). Due to the strong involvement of TRPV1 in the transmission of pain processes, structure-activity studies allowed for modifications in the structure of capsaicin and the development of analogous compounds in the attempt to eliminate the undesired irritation effect and pungency (Baskaran et al., 2018).

In this context, numerous specific TRPV1 antagonists have been developed with the aim of inhibiting nociceptive sensitization and the resulting transduction of the pain signaling process (Norman et al., 2007). Capsazepine was described as the first competitive antagonist of the channel, but its poorly selective binding capacity made it fail in clinical trials (Bevan et al., 1992). In models applied to the treatment of dental pain, examples of clinical records of compounds and their respective laboratories include AMG 517 (Amgen), AZD1386 (AstraZeneza), MK2295 (Merck/Neurogen) and SB705498 (GSK) (Trevisani and Szallasi, 2010). The clinical trial coordinated for SB705498 described it primarily as a powerful *in vitro* TRPV1 antagonist with the ability to reverse inflammation and pain *in vivo* (Gunthorpe et al., 2007). The controlled clinical trial was a multicenter (Korea, Italy, and UK), single-blind and placebo randomized study with a total of 145 patients, and the evaluated outcome corresponded to postoperative pain during third molar extraction surgery (Gunthorpe et al., 2007; De Petrocellis and Moriello, 2013). The experimental groups consisted of the treatments: SB705498 (400 mg and 1,000 mg), placebo and co-codamol (2 capsules of 500 mg paracetamol, 12.8 mg phosphatized codeine and 2 placebo capsules) (Cairns, 2009). In January 2019, the phase II results concluded in 2008 were published and no serious adverse effects were found; however, reported effects of SB705498 included headaches (n = 5) and heat sensation (n = 2) for the highest dose of 1,000 mg (Gunthorpe et al., 2007). The pharmaceutical industry has made a concerted effort to generate potent and selective compounds for TRPV1, in particular due to its attractive mechanism of action, which differs from the nonsteroidal anti-inflammatories, and also in the attempt to find a drug with a wide therapeutic window (Cheung et al., 2008; Trevisani and Szallasi, 2010).

## Novel Approaches for Dental Pain Treatment and Beyond: Regenerative Medicine and Others

**Figure 2:**
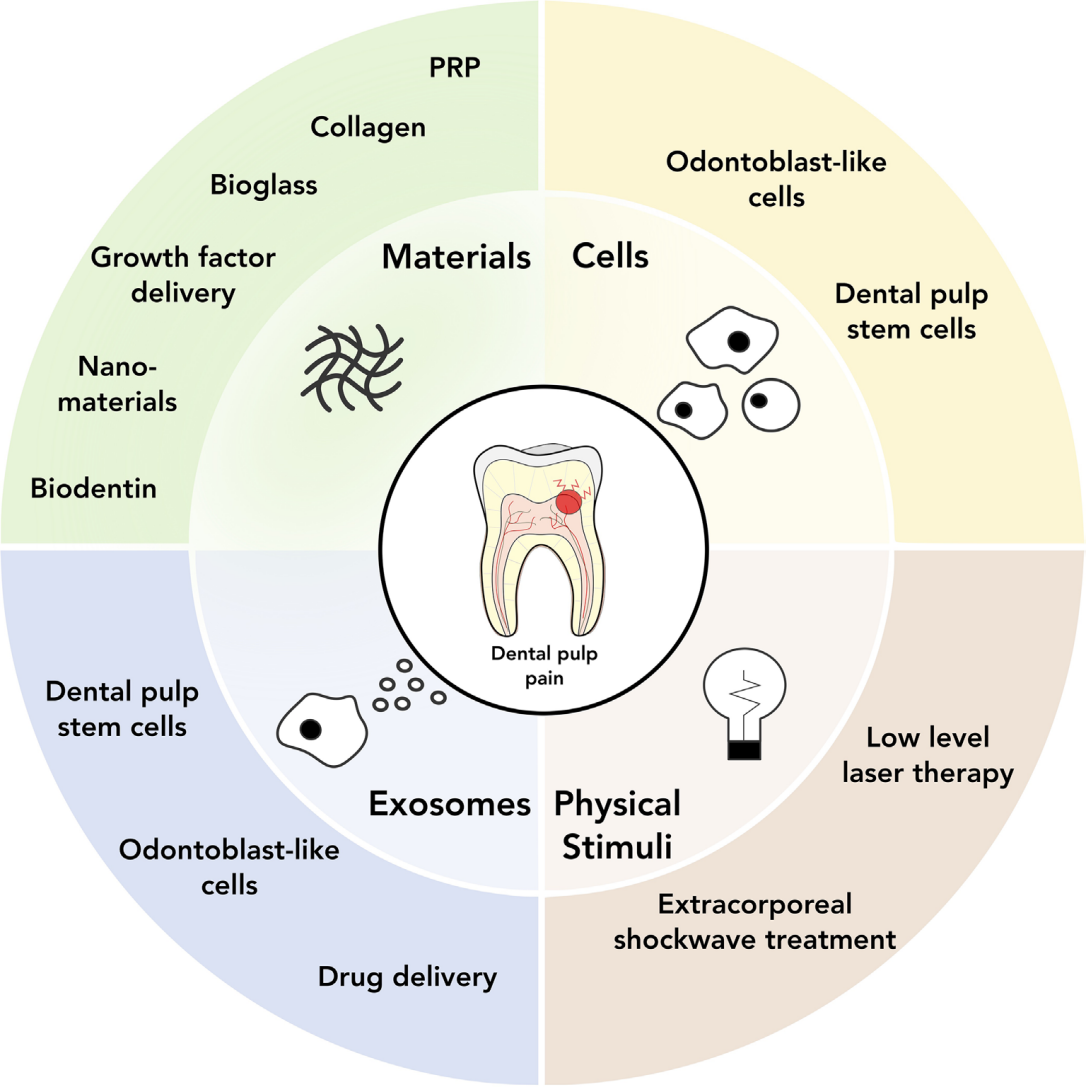
Overview of current and potential regenerative medicine approaches for dental pain treatment.

Regenerative medicine, and more specifically cell and tissue engineering approaches, have become increasingly popular in dental research over the last years. The key concept underlying these approaches is to develop a causative treatment aiming for long-term regeneration rather than immediate pain control. Due to its complex structure, regeneration of dentin and dental pulp has been proven a difficult and versatile task, which can be firstly categorized according to the primary clinical problem tackled (antibacterial treatment or pulp restoration) and subsequently into the following strategies: a) bio(active)materials, b) stem cells and cell-based approaches, c) exosomes, and d) physical stimuli, as well as combinations of the above approaches (**Figure 2**).

### Bioactive Materials

The use of active materials to control inflammation, infection and pain has been described since ancient times. Ancient Egyptians utilized pastes of ochre, honey, and willow extract (containing salicin) to treat loosening teeth as well as dental pain (Leek, 1967; Forshaw, 2009). The activity attributed to the materials used is mostly antibacterial, with salicin also displaying anti-inflammatory properties. Utilizing bioactive materials is a strategy pursued until today, currently aiming to ensure bio-and cyto-compatibility as well as reliable mechanic properties. Bioactive materials extensively used for pulp capping have been *calcium hydroxide* (causing mineralization by reduction of pyrophosphate) (Farhad and Mohammadi, 2005), *mineral trioxide aggregate* (MTA) (Torabinejad and Chivian, 1999) (a Portland cement-based substrate stimulating proliferation, migration, and differentiation of dental pulp stem cells into odontoblast-like cells to produce a collagen matrix) (Parirokh and Torabinejad, 2010; Paranjpe et al., 2011) and *biodentine* (a tricalcium-silicate based susbtrate with similar properties as mineral trioxide aggregate in terms of dental pulp stem cell differentiation) (Luo et al., 2014; Malkondu et al., 2014). Newer materials based on bioglass derivates are aiming to improve overall results of pulp capping, however, so far have only demonstrated decreased setting times and increased compressive strength as main favourable characteristics (Long et al., 2018; Hanada et al., 2019). Addressing the issue of biofilm formation, Yang et al. added the antimicrobial quaternary ammonium salt monomer 2-methacryloxylethyl dodecyl methyl ammonium bromide to Portland cement, and could demonstrate a *Streptococcus mutans*-inhibiting effect for up to 6 months (Yang et al., 2014). Kwon et al. (2019) used 2-methacryloyloxyethyl phosphorylcholine in combination with mineral trioxide aggregate and found a two-fold effect: on the one hand the composite demonstrated protein-repellent properties and prevented adhesion of *Enterococcus faecalis*, and on the other hand increased calcium ion deposition on the surface compared to mineral trioxide aggregate alone.

With recent advances in nanoscale manufacturing, an increased number of approaches have utilized the opportunities given by nano-structured materials to effectively deliver drugs into the dental pulp. Cuppini et al. (2019) developed a tricalcium-phosphate based paste containing amoxicillin microspheres, calcium tungstate and indomethacin nanocapsules to deliver antimicrobial and anti-inflammatory compounds to the defect site. Zhu et al. (2017) designed calcium silicate nanoparticles in combination with silver and zinc as a nano-disinfectant for dentin tubules and root canals. However, Ji et al. (2018) pointed out a limitation of this approach as insufficient amounts of nanoparticles are transported reliably through dentinal tubules; thus, they designed magnetic nanoparticles for directed transport through the dental pulp. Iron nanoparticles coated with prednisolone were applied onto the dental pulp and directed through dentinal tubules using external magnets, and authors hypothesized that this approach can serve to transport medications to reduce dental pulp inflammation, as well as enhance bond strength of composite resin to dentin (Ji et al., 2018).

Another potential way of controlling pain related to dentinal hypersensitivity is to develop materials that promote dentinal regeneration. As type-I collagen is the most abundant structural protein in the extracellular matrix, collagen-derived scaffolds have been of interest to promote homing and differentiation of cells for dental pulp regenerative purposes (Prescott et al., 2008). To induce reparative dentinogenesis, Rutherford et al. (1993) utilized osteogenic protein 1 and bone morphogenic factor 7 in a collagen matrix, as a chemo-attractive layer on dental pulp sealed with temporary dental cement. In the case of osteogenic protein 1, a higher amount of dentin deposition was observed while bone morphogenic factor 7 did not induce reparative dentinogenesis (Rutherford and Gu, 2000). Using a similar approach involving a combination of growth factors (bFGF, VEGF, or PDGF with a basal set of NGF and BMP7), Kim et al. (2010b) highlighted a beneficial effect of applying growth factor cocktails to stimulate cell homing and subsequent expression of van Willebrand factor, dentin sialoprotein, and NGF. Also, Rosa et al. (2013) found that an injectable type I collagen scaffold in combination with stem cells from human exfoliated deciduous teeth (SHED) was able to express odontoblastic differentiation markers when implanted into root canals. The resulting pulp-like tissue presented similar vascularization and cellularity compared to control dental pulps, and formation of new dentin was observed throughout the root. Furthermore, Jiang et al. (2017) induced root canal regeneration of immature teeth by using a resorbable collagen membrane paired with MTA, and observed complete resolution of signs and symptoms in all patients. However, no statistically significant improvement in regeneration was observed compared to the MTA-only control group. Besides collagen, other materials have also been of interest for promoting pulp regeneration, such as platelet rich derivates such as platelet rich plasma (PRP) or platelet rich fibrin (PRF). These materials are known for their richness in growth factors, which makes them a popular biomaterial in several areas of regenerative medicine (De Pascale et al., 2015). Regarding dental pulp regeneration, several groups have utilized the strategy of autologous PRP/PRF with varying results. While Torabinejad et al. (2015) and Rodríguez-Benítez et al. (2015) reported improved rates of bone formation and re-vascularization, Bezgin et al. (2015) found no advantages in using PRP for pulpal repair. Overall, further research is necessary in order to clarify the potential effectiveness of PRP and PRF for dental pulp regeneration.

It is important to note that these material-based approaches rely mainly on the regenerative capacity of the dental pulp, augmenting the formation of new dentin by stimulating tissue-resident dental pulp stem cells and controlling inflammation as well as infection. In aging teeth however, dentinal regeneration with material-based approaches has significantly lower success rates (Lipski et al., 2018), creating a demand for combined cell-material approaches.

### Cell-Based Approaches

The dental pulp is a tissue rich in vasculature and different cell types such as fibroblasts, dental pulp stem cells (DPSC), and odontoblasts. The latter are capable of forming dentin by production of collagenous and non-collagenous mineralizing proteins (known as secondary dentin). Furthermore, the odontoblast layer is located at the interface between dental pulp and dentin, protruding their processes into the dentinal tubules (**Figure 1B**). The dental pulp is crucial for tooth homeostasis and presents stimulus-dependent strategies for self-repair known as reactive dentinogenesis, where stimuli such as dental caries can induce odontoblasts to deposit tertiary dentin (Smith et al., 1995). Reparative dentinogenesis involves the dental pulp and its cellular components in response to a severe insult. When the odontoblast layer is disrupted, DPSC migrate into the site of injury and differentiate into odontoblast-like cells, forming new but irregular and less tubular dentin (Mitsiadis and Rahiotis, 2004; Tziafas, 2010). The presence of DPSC therefore is crucial for reparative dentinogenesis to occur. However, with age the dentin layer becomes thicker and the pulp chamber decreases in size, limiting the amount of DPSC available for regeneration. Furthermore, the resident DPSC are subjected to senescence through the p16INK4a/Rb pathway, driving the otherwise highly proliferative cells into cell cycle arrest (Feng et al., 2014). Hence, the main goals in pulpal cell therapy are to deliver DPSC to the damaged, resected or retracted pulp chamber, and/or to transplant odontoblasts to obtain dentin restoration.

DPSC display common features to mesenchymal stem cells such as ability to differentiate into several mesenchymal lineages, as well as a low immunogenicity. The latter makes them suitable for allogeneic stem cell therapies (Pierdomenico et al., 2005). Several studies speculate whether odontoblast-like cells differentiated from DPSC display the same characteristics as native odontoblasts (Shi et al., 2005; Huang et al., 2009). In the early 2000s, proof-of-concept studies demonstrated a high potential of dental-derived stem cells to differentiate into a functional pulp-like complex, using hydroxyapatite in ectopic rat models (Gronthos et al., 2002; Miura et al., 2003). Interestingly, in a similar model using 3D printed hydroxyapatite and DPSC or apical papilla stem cells, Hilkens et al. (2017) could not find significant differences between the stem cell- and cell free-groups in terms of angiogenesis. However, it was shown by Huang et al. (2010) that apical papilla stem cells and DPSC are capable of producing pulp-like tissue *de novo*, differentiating into odontoblast-like cells producing dentin-like tissue on existing dentinal walls.

One of the key questions in cell therapy is the route of delivery through which cells are administered to the site of interest, with approaches ranging from free cell transplantation to scaffold-free cell sheets and natural or synthetic scaffolds. Collagen and atelocollagen served as scaffolds to deliver mesenchymal stem cells in several studies (Iohara et al., 2011; Iohara et al., 2013; Hayashi et al., 2015), improving the formation of pulp-like tissue and secondary dentin in dog models (Iohara et al., 2011; Iohara et al., 2013). In a pilot clinical study, functional dentin formation was observed with no adverse effects (Nakashima et al., 2017). Interestingly, DPSC transplantation with platelet-rich fibrin did not show superior results compared to controls and was found to produce mainly periodontal tissue and not pulp (Hausner et al., 2007). Chitosan-hydrogel scaffolds seeded with DPSC in combination with growth factors formed pulp-like tissue and a layer of regenerative dentin in a necrotic-tooth dog model (El Ashiry et al., 2018). Decellularized swine dental pulp seeded with DPSC was found to induce the formation of pulp-like tissue and a layer of odontoblast-like cells in a subcutaneous mouse model (Hu et al., 2017). Also, Zhang et al. (2017) analysed decellularized tooth bud scaffolds seeded with porcine dental epithelial cells, human dental pulp cells, and human umbilical vein endothelial cells in combination with BMP-2, and discovered formation of organized dentin in a mini-pig model. Furthermore, in a scaffold-free approach, Itoh et al. (2018) designed a dual-celltype sheet containing undifferentiated DPSCs and differentiated odontoblast-like cells, reporting pulp regeneration in an ectopic mouse model. Xuan et al. (2018) demonstrated in a minipig–pulpectomy-model that pelleted DPSC are capable of regenerating whole pulp tissue, including the odontoblast layer. In a subsequent clinical trial, they observed similar effects in patients and furthermore sensory nerve regeneration.

### Exosome Therapies

One of the main recurrent issues in cell therapy is promoting the survival of transplanted cells in the recipient tissue over time. Factors like mechanical and nutritional stress, hypoxia, or detachment from the extracellular matrix as well as immune responses limit cell survival during and after the transplantation process. Many attempts have been made to increase cell homing and design long-term active cells therapies including biomaterial- or growth factor co-administration, reactive oxygen species protected hydrogels or preconditioning of the cells, among others (reviewed by Baldari et al., 2017). Within this context, exosomes have been proposed as an interesting alternative to cell transplantation and have gained popularity over the last decade, avoiding the challenge of cellular homing. Exosomes were first described in mammalian cell culture in 1981 as the cargo of microvesicles being shed from cells (Trams et al., 1981). They were believed to be a cellular “waste” system, mainly involved in the removal of obsolete molecules from the cell and cell wall (Pan and Johnstone, 1983). Due to key findings such as their role in expression of antigens (Raposo, 1996) and ability to transfer mRNA/miRNA (Valadi et al., 2007), they are now established as one of the main factors in inter-cellular communication. By definition, exosomes are vesicles between 30 and 150 nm in size, and their cell surface markers and cargo can be associated to the phenotype, metabolic status, and biological role of their cell of origin (Ratajczak et al., 2006; Valadi et al., 2007; Alcayaga-Miranda, 2016).

Exosomes have been harvested from several types mesenchymal stem cells and have demonstrated immune-modulatory and pro-angiogenic properties, amongst others (Liang et al., 2016; Cosenza et al., 2018). Within the analysed sources, DPSC have been demonstrated to produce exosomes with the capacity of regulating acute inflammation by suppressing activities of cathepsin B and matrix metalloproteinases in a carrageenan-induced inflammation model (Pivoraitė et al., 2015). As mentioned above, exosomes are a tool for cellular communication, representing the cell’s current state. Huang et al. (2016) utilized this characteristic and hypothesized that cell-type specific exosomes can trigger lineage-specific differentiation of stem cells. They differentiated DPSCs into odontoblast-like cells to subsequently isolate the exosomes and could verify a superior odontoblast differentiation *in vitro* as well as regeneration of pulp-like tissue in an *in vivo* tooth slice model. Hu et al. (2019) confirmed these findings and analysed underlying mechanisms, finding an activation of the TGFβ1/smads signalling pathway *via* transfer of microRNAs.

Aside from active native cargo, exosomes have shown to be promising system for bio-inspired and targeted drug delivery (reviewed by Zhang et al., 2019). Despite a lack of studies reporting the use of drug-loaded exosomes for the treatment of dental pain, there are several likely possibilities originating from experiences in other tissues. One potential avenue could be the local delivery of anti-inflammatory drugs into the pulp, as curcumin-loaded stem cell or macrophage exosomes have shown to display anti-inflammatory properties (Sun et al., 2010; Kalani et al., 2016). Also, delivery of liposome-encapsulated superoxide dismutase and catalase has demonstrated to locally suppress periodontal inflammation in a dog model (Petelin et al., 2000). Alternative approaches, originating from advances in nano-engineering, have demonstrated that exosomes can also be directed to specific tissues, making them an attractive option for targeted therapy (Gomari et al., 2018; Tian et al., 2018). Furthermore, several groups have designed artificial ion channels using DNA origami (reviewed by Shen et al., 2014). These channels could potentially resemble the biologic function of channels (i.e. TRP channel) and could be inserted as “mock-channels” into cell membranes. Overall, exosomes appear to be a promising alternative to cell therapy for dental pulp pain treatment; however, their efficacy relies strongly on recipient cells, which could be an issue in cases where the dental pulp is necrotic or a pulpectomy has been performed.

### Physical Therapies

Aside from pharmacological pain treatment or causative tissue engineering approaches such as biomaterials and cell-based therapies, physical therapies such as photo-biomodulation or shockwave treatment are an upcoming field in dentistry. Some studies have tried to modulate pain and inflammation with these approaches, including treatment of dental pain.

Low level laser therapy (LLLT) was invented in the 1960s, and in an attempt to identify cancer risks of laser it was found to induce hair growth in mice (Mester et al., 1968). It utilizes wavelengths between 600 nm and 1,000 nm and has been found to trigger many effects at the cellular and tissue levels. For example, cytochrome C oxidase absorbs near-infrared light, triggering downstream events such as changes in ATP, reactive oxygen species and nitric oxide (reviewed by Farivar et al., 2014). Regarding dental pain management, LLLT has been reported to reduce pain in several situations such as postoperatively after root canal retreatment (Arslan et al., 2017) or in cases of dentinal hypersensitivity (Gerschman et al., 1994; Orhan et al., 2011). However, several studies also demonstrate a regenerative effect of LLLT. In an *in vitro* tooth-slice model, LLLT increased odontogenic and angiogenic gene expression (El Nawam et al., 2019). Kim et al. (2018) also reported increased dentinogenic differentiation of DPSC *in vitro* using pulsing LLLT, proposing high production of reactive oxygen species and activation of the TGF-β1 signalling pathway as potential mechanisms. In rat models it was shown that LLLT increased the density of odontoblasts and induced formation of more regular dentin after mechanical pulp damage (de Santana et al., 2017) and furthermore, stimulated cell proliferation as well as dentin formation after pulpal apoptosis (Shigetani et al., 2011). These results suggest LLLT as a promising non-invasive approach for dental pain management in diverse clinical situations, although further studies are needed to confirm its clinical reproducibility.

Extracorporeal shockwave treatment (ESWT) has demonstrated beneficial effects in several areas of regenerative medicine [e.g. bone regeneration (Schaden et al., 2001) or ischemia-induced tissue necrosis (Mittermayr et al., 2011)]. Shockwaves are sonic pulses characterized by an initial rise in pressure, reaching a positive peak of up to 100 MPa within 10 ns, followed by a negative amplitude of up to 10 MPa and a total life cycle of less than 10 µs (Ogden et al., 2001). The underlying effect on cells and tissues has been attributed to increased ATP secretion and subsequent activation of the extracellular-signal-regulated kinase pathway (ERK) (Weihs et al., 2014). For DPSC it has been demonstrated that extracellular ATP activates P2 receptors and downstream signaling events that induce odontogenic differentiation, with ESWT suggested as one of the triggering factors (Wang et al., 2016). Furthermore, it has been shown that ESWT increases efficacy of desensitizing agents for dentine hypersensitivity (Datey et al., 2016). Despite these promising initial studies, there remains a lack of animal and clinical studies associating ESWT to dental pain control, and future research is needed to strengthen these initial observations.

## Conclusions and Future Perspectives

Although significant improvements have been made in dental care, dental pain remains a frequent source for patient poor quality of life. Current treatment is mainly centered around a combination of the pharmacological management of symptoms and restorative treatment, nonetheless drugs such as NSAIDs or opioids can potentially present significant side effects and addiction in limited patients. Thus, there are many novel approaches for dental pain management being explored in hopes of improving treatment outcomes in the long-term. Approaches such as TRPV1-targeting drugs have shown promising results so far, although further clinical research is needed in order to demonstrate their efficacy in dental pain management. Furthermore, causative treatment approaches originating from regenerative medicine have the potential to significantly improve the resolution of dental pain. Cell-based and exosome therapies are already being used in other fields, and introduction into clinics could provide long-term solutions for the modulation of local pulp inflammation and nociception. Material-based therapies that promote tooth remineralization have been used in the past, and current advances improving bioactivity and antibacterial effect of materials could provide clinicians with novel approaches for the treatment of dental caries and subsequent dental pain.

Interestingly, physical therapies such as LLLT and ESWT have been widely used for the treatment of inflammation and pain in a range of tissues in humans (Cotler et al., 2015; Lee et al., 2017; Moya et al., 2018). One of the main advantages of these approaches is their non-invasiveness, generating a biological effect in deep tissues without the need of surgery of restorative treatment. Initial studies regarding use of LLLT and ESWT for dentinal hypersensitivity showed promising results (Gerschman et al., 1994; Datey et al., 2016), as well as the use of ESWT for controlling inflammation in other areas of the mouth. However, there is still a lack of clarity regarding the biological mechanism of ESWT in tissues. Further studies elucidating the mechanisms underlying ESWT as well as effectiveness to resolve dental pulp inflammation and pain are necessary.

As discussed throughout this review, teeth are highly specialized organs with a quite particular nociception mechanism. Nerve fibers within the dental pulp are triggered either by environmental factors (dentinal hypersensitivity) or by the release of pro-inflammatory and bacterial molecules. Thus, the ultimate goal of any therapy should be to reduce local inflammation while restoring protection to dentin and the dental pulp, in order to provide causative treatment and long-term success to patients. Current research should therefore focus on developing novel and causative treatments against dental pain, with limited adverse effects and clinically reproducible results.

## Author Contributions

CS and BB contributed equally to this manuscript. CS, BB and SA participated in the conception of the review. SA drafted the initial full manuscript. CS, BB and SA wrote and edited the manuscript.

## Funding

This work was sponsored by Fondo Nacional de Desarrollo Científico y Tecnológico (FONDECYT) grant #11180101. CS is supported by FONDECYT grant #11180406. BB is supported by FONDECYT grant #3170518. SA is supported by FONDECYT grant #11180101. MiNICAD is supported by Iniciativa Científica Milenio, Ministry of Economy, Development and Tourism, Chile.

## Conflict of Interest Statement

The authors declare that the research was conducted in the absence of any commercial or financial relationships that could be construed as a potential conflict of interest.
